# Empowering women in decision-making about mobility during labor: Insights from experts

**DOI:** 10.18332/ejm/205673

**Published:** 2025-07-29

**Authors:** Marlene I. Lopes, Margarida Vieira, Alexandrina Cardoso

**Affiliations:** 1 Health Sciences Research Unit, Nursing School of Coimbra, Coimbra, Portugal; 2 Universidade Católica Portuguesa, Centre for Interdisciplinary Research in Health, Faculty of Health Sciences and Nursing, Porto, Portugal; 3 Nursing School of Porto, CINTESIS Center for Health Technology and Services Research, Porto, Portugal

**Keywords:** prenatal education, nurse-midwives, childbearing women, decision-making, active birth, upright positions

## Abstract

**INTRODUCTION:**

Allowing freedom of movement and positions during labor facilitates safe progression, improves maternal and neonatal outcomes, and contributes to a positive birth experience for women and their partners. Childbirth preparation aims to empower women by equipping them with skills and confidence to manage labor actively and knowledgeably. However, in many hospital settings, women’s mobility remains restricted. This study explores expert perspectives on the core components of an intervention designed to promote women’s autonomy and active participation during labor, to be implemented by nurse-midwives within Childbirth Preparation Programs in primary healthcare.

**METHODS:**

A qualitative study was conducted in February 2024 in Portugal, using a focus group composed of ten nurse-midwives recognized as experts in childbirth preparation. The session was recorded and analyzed through content analysis, guided by the Theory of Emancipated Decision-Making in Women’s Healthcare.

**RESULTS:**

Nine women and one man participated, aged 34–64 years, representing primary healthcare, hospitals, and academia. Six key themes emerged from the analysis: the intentionality behind the intervention; understanding the meanings and expectations women hold about their birth experience; empowering women by providing knowledge and skills; raising awareness of social norms that may influence women's decisions and actions; encouraging women to reflect on their options; and fostering woman-centered care.

**CONCLUSIONS:**

The experts emphasized the importance of promoting mobility during labor to enhance perinatal outcomes and the childbirth experience. Empowering women through experiential learning, fostering reflection, supporting informed choices, and ensuring continuity of care through collaborative professional practices were identified as essential strategies for effective intervention.

## INTRODUCTION

A positive childbirth experience is a fundamental aspiration for pregnant women^1^, given its lasting emotional and psychological impact. It significantly shapes a woman’s self-perception as a mother, partner, and individual^2^. Childbirth preparation plays a central role in this process, enhancing women’s confidence and equipping them with the knowledge and skills to navigate labor effectively. Making informed use of available resources can contribute to better health outcomes and more meaningful birth experiences. Childbirth is influenced by various factors, including personal expectations, the quality of support received, the nature of the relationship with healthcare providers, and the degree of involvement in decision-making^3^. Active participation and a sense of control are particularly influential in how women perceive childbirth. Many women seek a birth experience aligned with their personal, cultural, and social values^4^. Although unpredictable, labor is often approached with a desire for physiological progression and autonomy. Control may be internal, managing pain, emotions, and choosing positions, or external, involving shared decisions about clinical care^4^.

Upright positions and mobility during labor are associated with multiple physiological and psychological benefits: enhanced comfort, improved fetal descent, reduced pain, more efficient contractions, and better maternal-fetal circulation^5^. Supporting women in adopting positions that feel right for them is therefore essential^1,5^. However, this requires women to feel confident and empowered to make informed, evidence-based choices.

Rising intervention rates and declining physiological births have raised concerns about their impact on maternal and neonatal health^6^. Even in low-risk pregnancies, care is often guided by a risk-averse and paternalistic model, where information may be selectively provided^7^. Some women report feeling pressured into accepting interventions or find providers to be hurried and emotionally unavailable, which can compromise their autonomy^8^. Despite participating in childbirth preparation, many women struggle to apply what they have learned, often due to a lack of support or conducive conditions for movement during labor^9^. These findings emphasize the need for comprehensive preparation that empowers women to make autonomous, informed decisions during labor, thereby preserving their self-determination and improving their birth experience.

To address this challenge, an intervention is currently being developed to support women in making informed decisions regarding movement and positioning during the first stage of labor. Designed within the MRC Framework for Complex Interventions^10^, the program will be delivered by nurse-midwives in primary healthcare settings. Stakeholder engagement during the development phase of complex interventions is essential to ensure both feasibility and impact. A preliminary study explored the context and perspectives of nurse-midwives responsible for implementing the intervention in UCC^11^, as well as the views of the women who received it.

This study presents expert insights into childbirth education to help identify the key components of the proposed intervention.

## METHODS

### Design

A qualitative study was conducted with expert nurse-midwives to explore their experiential knowledge and insights regarding the target population and the context of childbirth education programs within primary healthcare settings. The focus was specifically on mobility and upright positions during the first stage of labor. Their expertise aimed to inform the operationalization of the intervention, tailoring it to the women’s needs and the realities of clinical practice. Participants were selected through purposive sampling, based on the following inclusion criteria: demonstrated experience in clinical, managerial, or academic roles related to the design, implementation, and/or evaluation of interventions that promote women’s empowerment in physiological childbirth and involvement in decision-making. One focus group was conducted in February 2024. The study followed the COREQ guidelines for reporting qualitative research^12^.

Ethical approval was obtained from the Health Ethics Committee of the Centro Regional Health Administration on 22 June 2023 (No. 52/2023). Participants received comprehensive information about the study, were informed of their right to refuse or withdraw at any time, and provided informed consent through an online form.

### Setting

The study took place in Portugal, which has a universal public healthcare system led predominantly by physicians and obstetricians. Women have free access to healthcare throughout pregnancy, childbirth, and the postpartum period. Pregnant women are encouraged to attend childbirth preparation programs conducted by nurse-midwives in Community Care Units (CCUs) within the primary healthcare system. Labor wards, overseen by obstetricians, differ in their policies regarding mobility and upright positions during labor. Nearly all births (99.75%) occur in hospital settings, typically attended by obstetricians or nurse-midwives, with an average hospital stay of two days for mothers and newborns.

### Sampling, participants, and recruitment

Fourteen nurse-midwives, recognized as experts in childbirth education and experienced in supporting laboring women in both primary healthcare units and hospital delivery rooms, were invited via email. To ensure representativeness and account for geographical, rural–urban, and cultural diversity, purposive sampling was used to recruit participants from various regions of Portugal. The research team defined the eligibility criteria and purposively selected nurse-midwives who demonstrated the capacity to implement the intervention in a future feasibility study. Ten nurse-midwives confirmed their availability and consented to participate.

### Data collection

The focus group was conducted online via Zoom by two researchers, at a time convenient for participants. This approach facilitated the inclusion of nurse-midwives from across the country and avoided travel-related barriers. Prior to the session, all participants completed an online form providing sociodemographic data, including age and previous experience in childbirth education. The discussion followed an interview guide developed by the research team (Table 1). The session was recorded in both audio and video formats, and the principal researcher transcribed the interview verbatim immediately after the session.

**Table 1:** Guiding questions used in the focus group with expert nurse-midwives in Childbirth Education

*Questions*
1. Do you believe there are specific care needs to empower women to make decisions about their mobility and upright positions during labor? If so, what are these needs?
2. Based on these needs, what is the therapeutic intention behind nurse-midwives implementing such interventions?
3. What content and strategies are essential to effectively empower women to make informed decisions about their mobility and upright positions during labor?
4. What conditions must be met to ensure the successful implementation of this intervention?

### Data analysis

Data from the focus group were analyzed using content analysis^13^. The process was informed by the conceptual model of the Emancipated Decision-Making Theory in Women’s Healthcare, which highlights the role of societal norms in shaping, and at times constraining, women’s autonomy in health-related decision-making. This theory posits that women may feel pressured to align with socially accepted norms rather than make choices that best reflect their individual needs, often leading to dissatisfaction. Emancipated decision-making encompasses five core dimensions: 1) access to evidence-based knowledge; 2) critical reflection on traditional or authoritative practices; 3) recognition of personal knowledge and needs; 4) awareness of tensions between individual desires and societal expectations; and 5) the creation of supportive environments that allow women to make autonomous decisions without guilt or negative consequences. Promoting autonomy in decision-making has been associated with greater satisfaction, confidence, and maternal well-being^14^.

The transcripts were read multiple times and cross-checked with the video recordings before coding. Notes were taken throughout the process, and recurring themes and patterns were identified to develop an initial coding framework. NVivo software was used to assist in managing and organizing the data. Themes and categories were refined and finalized through iterative discussions within the research team.

## RESULTS

The study engaged ten expert nurse-midwives with experience in childbirth education: seven from Community Care Units (CCUs), two from hospital delivery rooms, and one from a Nursing and Midwifery School. Among them, nine were female and one was male, aged 36–64 years with average age 49.1 years (SD=9.3). Their experience in nurse-midwifery varied from 10 to 34 years, with an average of 18.0 years (SD=7.9). These participants came from five different districts across the northern and central regions of Portugal. The duration of the focus group session was 2 hours and 36 minutes.

### Main themes

**Figure 1:**
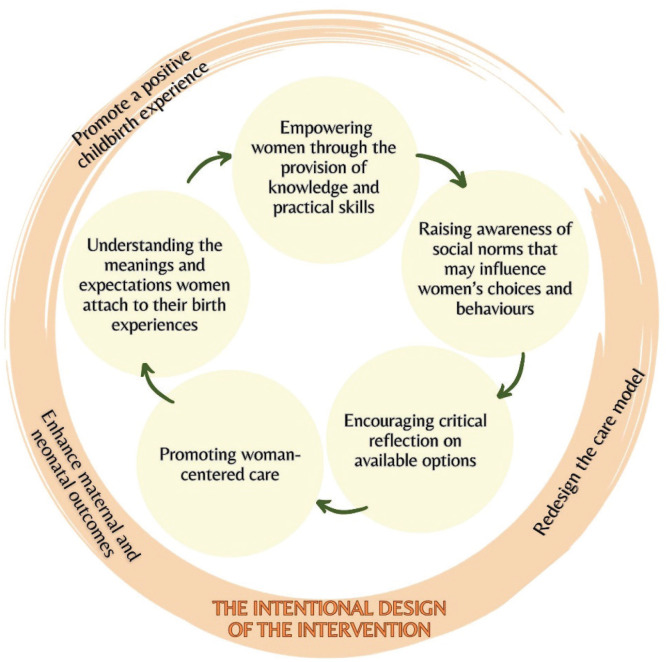
Main themes identified by expert nurse-midwives to inform an intervention supporting women’s decision-making on movement and positioning during the first stage of labor

Six main themes emerged from the expert discussions: 1) The intentional design of the intervention; 2) Understanding the meanings and expectations women attach to their birth experiences; 3) Empowering women through the provision of knowledge and practical skills; 4) Raising awareness of social norms that may influence women’s choices and behaviors; 5) Encouraging critical reflection on available options; and 6) Promoting woman-centered care. Figure 1 provides a schematic representation of these interconnected themes.

*The intentional design of the intervention*

Experts unanimously agreed that empowering women to remain active during labor is a crucial element of childbirth preparation, with significant benefits for both maternal and neonatal outcomes. Promoting mobility is also seen as central to fostering a positive birth experience. Labor is understood as a unique and transformative moment in a woman’s life, making it essential for her to feel in control, confident in expressing her needs, and capable of managing the process effectively. In this context, freedom of movement and positioning is considered key to achieving these goals:

*‘The woman should be able to use strategies, decide when to apply them, and determine the appropriate moment ... having the perception that she can choose when and what to use, and the correct way to do so at each stage.’* (P4)

Experts also emphasized that promoting active labor contributes to the prevention of complications related to immobility and helps women manage labor pain more effectively. Free mobility is therefore viewed not only as a right but also as a health-promoting measure:

*‘To minimize, through the benefits of mobility, the consequences that can arise from total immobility during labor, whether in the first stage or the delivery itself.’* (P5)

*‘It could also aim to reduce pain.’* (P8)

Two participants further suggested that when women are empowered to make informed choices and recognize the benefits of mobility for themselves and their babies, they may drive a broader shift in maternity care practices. By advocating for more supportive environments, these women can influence healthcare systems to better align with their expectations:

*‘In the long term, I believe we could see a societal shift among women, as changes often stem from the demands they bring to healthcare services. If women want to remain mobile and adopt upright positions, services will inevitably need to reorganize to provide this type of care, and we’re already seeing this happen in many places.’* (P6)

*Understanding the meanings and expectations women attach to their birth experiences*

All experts agreed on the importance of understanding the meanings and expectations women associate with childbirth, particularly in relation to their involvement in decision-making around mobility and upright positions. One expert highlighted the variability among women: while some are well-informed and actively seek an engaged role during labor, others show limited interest or readiness. These differences reinforce the need for individual consultations with each woman, and her partner, if desired, to tailor the approach to their unique context and preferences:

*‘I often encounter two extremes: women who are very informed and want more mobility during labor, and others who don’t want to think about it at all.’* (P8)

*‘The desire has to come from the woman; she needs to express it. We shouldn’t impose our will.’* (P1)

Three experts emphasized the importance of thoroughly assessing each woman’s expectations and perceptions of labor. They noted that many women hold idealized, and sometimes unrealistic, views of childbirth, often influenced by social media content that lacks clinical context. This can lead to a desire for total control and detailed planning, which contrasts with the unpredictable and dynamic nature of labor. The sources of information women rely on significantly shape their expectations; they can offer reassurance and promote preparation, but they may also create anxiety or disappointment when reality does not align with preconceptions:

*‘Today’s couples are not like those from a few years ago ... they are very informed, sometimes overly informed, absorbing both correct and incorrect information ... It’s our job to understand the type of information they’re bringing and help them clarify ... explaining what labor is and the advantages of upright positions. They are so connected to technology, they want everything planned and scheduled, even the birth. They come into the delivery room and ask, “What time is it going to happen?” ... We need to help them understand that labor, as the name suggests, is work, it’s a physical test, sometimes long, sometimes short, but always dynamic ... Couples focus on the internet, on a specific birth type, position, or technique, thinking it will work miracles, but everything is dynamic and must be adapted in the moment.’* (P5)

Experts also noted the importance of exploring previous childbirth experiences, as these significantly influence current expectations. Additionally, assessing a woman’s knowledge about mobility and upright positions, as strategies for facilitating labor progression and pain management, can provide valuable insight. One expert suggested asking women to describe or physically demonstrate how they envision using mobility during labor. This approach allows for a clearer understanding of their practical readiness:

*‘By asking the woman to describe how she would use mobility and upright positions during labor, or how useful she thinks they would be ... she could either describe it or, ideally, demonstrate it. Descriptions can sometimes be misleading, as the woman might not describe exactly how she would do it. By demonstrating, we can assess it ourselves by observing how she would actually apply these techniques.’* (P4)

*Empowering women through the provision of knowledge and practical skills*

Experts unanimously emphasized the importance of equipping women with evidence-based knowledge as a foundation for informed decision-making. Providing accurate, accessible information enables women to make choices that align with their expectations, preferences, and individual needs. In particular, it is crucial to clearly explain the relationship between mobility, upright positions, the physiological progression of labor, and improved maternal and neonatal outcomes:

*‘Informing women about recommended practices, the importance of mobility during labor, and upright positions, highlighting that it’s a World Health Organization recommendation.’* (P8)

Ongoing practical training in mobility and upright positions is essential to help women apply these techniques effectively during labor. Hands-on experience fosters the development of embodied knowledge through active exploration of movement, positioning, and the dynamics of pelvic mobility and adaptability. One expert emphasized that when women physically experience the effects of these movements in their own bodies, it fosters trust and strengthens their confidence. Feeling the benefits first-hand, not just being told about them, enables women to recognize which strategies work best for them, enhancing both self-awareness and autonomy during labor:

*‘It’s vital for women to feel the results in their bodies. The outcome is not told to them, it’s felt. This builds trust because they know the result is genuine, helping them choose strategies that work best for them. Even in a group setting, the experience is deeply personal.’* (P4)

Providing women with concrete strategies to remain active during labor is essential. Experts emphasized the importance of preparing women to manage early labor at home, where they typically feel more comfortable and in control. This autonomy can positively influence labor outcomes by promoting relaxation, reducing stress, and supporting the natural progression of labor:

*‘Women like step-by-step instructions, like a guidebook ... I always try to empower them to stay home longer and handle early labor there. I encourage them to do household tasks, clean windows, dance, or finish tidying up.’* (P10)

*‘Teaching them the right time to go to the hospital is crucial, it greatly affects the success of labor. Women can often complete a significant portion of labor at home, using mobility for comfort. Movement is intuitive.’* (P5)

One expert emphasized that, beyond explaining the physiological mechanisms of labor, it is equally important to reaffirm women’s inherent ability to give birth, a capacity historically recognized and trusted. Drawing on traditional wisdom can help normalize mobility during labor and foster confidence in the birthing process:

*‘In my grandmother’s and great-grandmother’s time, they used to say, “Go clean the house” when labor started ... this generation hasn’t heard that, so it’s important to explain why mobility matters.’* (P5)

Experts also acknowledged that feelings of fear and uncertainty during early labor at home are common among women and their partners. To mitigate anxiety and support decision-making, strategies should be implemented that offer practical, reassuring guidance. This can empower women to remain active and comfortable within their familiar environment, while avoiding unnecessary early hospital admissions:

*‘Couples are often afraid of being alone at home and go to the hospital too early when they could have stayed home safely. There should be guidelines to help them describe their feelings and receive advice, like “Take a shower, go for a walk, and call back in an hour” ... It’s different from what they learn in class, being alone at home makes them more anxious.’* (P5)

Alternative movements and positions that can be performed in bed, when full mobility is restricted, should also be addressed in childbirth preparation. These options should be practiced in advance, and women encouraged to request them from their nurse-midwives during labor. Even when upright movement is not feasible, simple adaptations such as elevating the head of the bed or adopting side-lying positions can still facilitate labor progression and promote comfort:

*‘For some women, we can’t promote full mobility due to fall risks, but there are alternatives like raising the head of the bed or side-lying positions. Even when upright movement isn’t possible, these techniques can still facilitate labor, and they should be encouraged in preparation programs.’* (P7)

Experts further emphasized that true empowerment requires addressing not only physical strategies but also the emotional dimension of labor. Promoting women’s self-efficacy through positive, affirming language and involving their partners as sources of support are considered essential to achieving a satisfying and autonomous birth experience:

*‘The mind has a huge influence on labor ... the emotional component is extremely important, and this starts in these programs. The challenge is motivating and empowering them, showing them they are capable.’* (P5)

To maximize the effectiveness of the intervention, experts recommended in-person sessions in small groups of four to six women and their partners. This format helps create a safe, informal environment that fosters trust, comfort, and open expression. It also encourages active participation and allows space for practical demonstrations:

*‘It’s important to build trust within the group. For women to feel comfortable, they need to know each other, and the group should be informal ... otherwise, they won’t engage in more intimate moments.’* (P6)

*Raising awareness of social norms that may influence women’s choices and behaviors*

Experts acknowledged that many women are guided not only by personal preferences but also by perceived social expectations, what they believe is ‘normal’ or appropriate behavior during childbirth. These expectations are often shaped by family members, healthcare professionals, and broader societal norms. This can generate pressure that limits women’s autonomy, comfort, and satisfaction with their decisions. For example, while many women feel confident discussing their questions and preferences during prenatal education, they often hesitate to present a written birth plan to hospital staff. Concerns about being perceived as demanding or disrupting professional routines may lead them to avoid formalizing their wishes:

*‘They’re very apprehensive about creating a birth plan ... they know they can express their emotions and ideals, but when it comes to handing over a document at the hospital, some are hesitant and prefer not to bring anything written, fearing it won’t be well-received.’* (P9)

On a more positive note, one expert observed a growing interest in birth plans since the introduction of an institutional template at a reference hospital. This initiative has helped normalize the topic, encouraging women to discuss their preferences both during childbirth preparation and hospital consultations:

*‘I’ve noticed an increasing interest in discussing and creating a birth plan. We now have an institutional plan at the hospital.’* (P3)

To support informed and realistic expectations, experts emphasized the importance of ensuring that nurse-midwives who lead childbirth preparation programs are familiar with hospital policies and practices. In the context of mobility and upright positions, women must be made aware that certain medical interventions, or the preferences of individual healthcare professionals, may restrict their freedom to move during labor. Providing this information allows women to make decisions based on a clear understanding of the conditions they may encounter, ultimately reinforcing their sense of agency and preparedness:

*‘I always maintain close contact with colleagues in the delivery room to stay updated on changes.’* (P10)

*‘In some maternity wards, as we’ve mentioned, they still administer epidural infusions and require the woman to remain lying down without moving ... Nowadays, there are more temporary anesthetists, and each follows different protocols.’* (P5)

*Encouraging critical reflection on available options*

Experts highlighted the importance of helping women navigate situations that may cause discomfort or diminish their confidence in making decisions aligned with their needs and values. It is essential to clearly present all available options regarding mobility and upright positions during labor. This information should be adapted to different contexts, whether at home during early labor or in the hospital setting, emphasizing how specific choices and medical interventions may influence their ability to move freely or remain upright:

*‘My approach is to give them strategies they can use before entering the delivery room, what they can do to maintain mobility and manage their timing for heading to the hospital, knowing that once there, their mobility might be more restricted.’* (P8)

Encouraging reflection on these options, and how each one may impact the woman physically, emotionally, and practically, can be facilitated through the use of simple, accessible tools such as flowcharts or visual summaries tailored to various labor scenarios. Group-based discussions using real-life or hypothetical situations can also serve as practical exercises in shared decision-making, helping women build confidence and anticipate possible challenges:

*‘We can create practical flowcharts or small cards for them to keep on hand, on their phones, with safety guidelines for staying at home and exercises to do ... We could also create scenarios based on real-life experiences, allowing them to make decisions together in groups.’* (P5)

*Promoting woman-centered care*

Experts agree that healthcare in all clinical settings should uphold women’s right to self-determination by supporting informed decision-making and providing appropriate, respectful care. However, as previously noted, they acknowledge that in some hospital environments, women may feel uncomfortable expressing their preferences and may face unnecessary interventions that limit their mobility. This disconnection frustrates not only the women but also the professionals who have invested time in preparing them for childbirth:

*‘I'm concerned that within the National Health System, this service isn’t equitable and, frankly, isn’t of high quality yet. There's no consistency in the level of care ... Not all delivery rooms provide opportunities that meet women’s expectations, which is very disheartening and limits our efforts. It's frustrating not to be able to guarantee that the right conditions are in place.’* (P6)

In response to these challenges, experts emphasized the need to actively promote woman-centered care by creating clinical environments where women feel safe, supported, and in control of their birth experience. Supporting women’s autonomy must be accompanied by systemic changes that ensure continuity of care across services. Experts advocated for regular communication between nurse-midwives working in CCUs, who lead childbirth preparation, and those in hospital labor wards, who provide intrapartum care. They highlighted the importance of understanding the realities of each setting and fostering collaboration to better align institutional practices with the expectations and needs of women:

*‘Teamwork between those in delivery rooms and those in primary care is essential. There should be meetings where we can discuss, “We're trying to implement this now, but women from these programs are struggling with this, while they’re improving in this area”. This feedback is crucial, I believe both sides must be willing to come together.’* (P5)

## DISCUSSION

The nurse-midwife experts participating in this study unanimously emphasized the importance of implementing childbirth preparation interventions that empower women to engage actively in labor. Freedom of movement and choice of birthing positions were consistently identified as key strategies to support labor progression, enhance maternal autonomy, and contribute to more positive birth experiences and improved maternal and neonatal outcomes. These findings align with the existing literature. A Cochrane review assessed the effects of upright versus recumbent positions, found that upright positions reduced the duration of the first stage of labor by approximately 1 hour and 22 minutes, lowered the likelihood of cesarean birth, and decreased the use of epidural analgesia^5^. Newborns of women who adopted upright positions were also less likely to require admission to neonatal intensive care. Moreover, freedom of movement during labor has been associated with greater maternal satisfaction, as women report feeling more involved in decision-making and experiencing higher levels of autonomy and self-efficacy^4,15^. In addition to its physiological benefits, movement serves as a coping strategy by offering comfort and distraction, which can reduce the perceived need for pharmacological pain relief. Recent studies have shown that dancing during labor increases maternal comfort and promotes a more positive experience^16^. Evidence also suggests that when women are supported to remain active, their partners tend to be more involved, contributing to their own sense of participation and emotional satisfaction^9,17^.

Beyond promoting positive birth experiences and improved perinatal outcomes, experts in this study expressed a desire to influence hospital care models by making them more flexible and responsive to women’s needs. Empowering women to make informed decisions about mobility during labor, based on personal preferences and evidence-based knowledge, was viewed as a means to challenge restrictive obstetric practices in certain settings. While women are not responsible for determining the appropriateness of clinical interventions, their expectations and questions may influence the attitudes and behaviors of healthcare professionals^18^. It is therefore important to examine whether women who are more informed and autonomous can contribute to shifts in institutional practices by advocating for care that is more individualized and aligned with their values. In this regard, nurse-midwives play a central role in fostering supportive care environments and promoting woman-centered approaches through their interactions with women during childbirth preparation^19^.

Encouraging women’s participation in healthcare decisions is a cornerstone of contemporary, rights-based maternity care. Women’s expectations regarding childbirth, and the outcomes they seek, are shaped by a complex interplay of personal experiences, social context, perceived vulnerability, and information from multiple sources. Experts in this study highlighted the importance of understanding each woman’s unique expectations and birth history to tailor the support provided. In cases where women show limited interest in decision-making or hold idealized, unrealistic expectations, often influenced by social media and other online sources, experts recommended reframing these perceptions to support more realistic and informed choices. This highlights the critical need for professionals to guide women in navigating digital resources. While many women find the internet helpful and reassuring, it can also increase anxiety, particularly when information is unfiltered or decontextualized^20^. Nurse-midwives must therefore provide guidance on the credibility and usefulness of online content, as many websites offer low-quality or misleading information^21^. Nonetheless, research shows that most women, regardless of educational or socioeconomic background, prefer face-to-face preparation. Attending in-person childbirth classes provides an opportunity to access reliable, evidence-based information, build confidence, and connect with other women, helping to normalize concerns and reduce feelings of isolation^22,23^.

Experts unanimously agreed that for women to make informed and autonomous decisions, they must be informed on the benefits of physiological childbirth and the evidence-based practices that support it, particularly the role of freedom of movement and positioning during labor. In addition to theoretical knowledge, experts repeatedly emphasized the value of first-hand experience, encouraging women to explore how movement affects pelvic dynamics and comfort. Repeated practice of these strategies is seen as essential to building meaningful, embodied knowledge and practical skills.

Constructive and interactive learning methods were highlighted as fundamental to this process, as they promote the internalization of new information and deepen understanding. In this regard, activities such as drawing concept maps, taking notes in one’s own words, or engaging in peer discussions contributed to increased knowledge, more positive attitudes, and stronger self-efficacy regarding mobility and upright positions during labor^24^. Improving childbirth self-efficacy through antenatal education has been linked to better perinatal outcomes^25^. Confidence in managing labor effectively influences women’s choices about delivery methods and increases their willingness to follow guidance from nurse-midwives, particularly when encouraged to adopt upright positions^26,27^.

Experts also underscored the importance of creating a welcoming, informal environment that fosters social connection among women as a key factor in building confidence. Vicarious learning, observing others who are perceived as similar, was described as a powerful mechanism for strengthening self-belief. This is especially relevant for nulliparous women, who benefit from seeing others with previous childbirth experience successfully engage in active labor strategies. Campbell and Nolan^26^ similarly observed that experienced mothers often adopt a protective and supportive role in group settings, voluntarily responding to questions from first-time mothers and offering reassurance based on lived experience.

The inclusion of birth partners and the use of positive communication were also identified as essential components of effective preparation. Experts highlighted the need for healthcare professionals to be aware of the language they use, as communication style can strongly influence women’s confidence and overall birth experience. Feeley et al.^28^ described midwives adopting a ‘non-bullying’ communication style, consciously avoiding negative phrasing such as ‘no’, and instead asking, ‘How can we achieve this?’. In contrast, Cutajar et al.^29^ found that childbirth preparation sessions often featured mixed messages, with both encouraging and discouraging statements, particularly in reference to the second stage of labor. Evidence suggests that positive, respectful communication fosters confidence and supports better outcomes, while negative or dismissive language can be counterproductive or even harmful^30^.

Empowering women to confidently manage early labor at home was highlighted by experts as a key strategy for promoting mobility and active engagement in labor. However, they acknowledged the emotional and practical challenges faced by women and their partners, particularly feelings of uncertainty and anxiety. Even when women feel they are coping well, many still present to the hospital prematurely due to a lack of confidence or a desire to share responsibility with healthcare professionals^31^. This decision is often influenced by the concerns of partners or family members, who may encourage early hospital admission as a form of reassurance. These feelings should be acknowledged and addressed during childbirth preparation. Experts stressed the importance of anticipating such scenarios by discussing them openly, offering practical, situation-specific guidance, and providing accessible resources in both digital and printed formats to support decision-making. Being available to clarify doubts when labor begins was also seen as crucial. Similar findings were reported by Butler^32^, who emphasized the value of helping women identify strategies to manage external pressures, from professionals, family, or friends, including the support of a trusted companion. Furthermore, it is essential to clarify the commonly used recommendation to ‘stay home as long as possible’, as it can be vague, difficult to interpret, and is not always well understood by women. Reducing the stigma associated with seeking care in early labor, and critically examining the notion of ‘false labor’, which may invalidate a woman’s experience, are also important. In addition, the implicit pressure to be the ‘right patient’ and to arrive at the hospital at the ‘right time’ warrants further exploration^33^.

Experts described the perception that women may experience subtle but significant pressure when making decisions about their care. One example is the varying levels of comfort women report when presenting a birth plan at the hospital, often influenced by whether the institution provides an official template. This highlights how perceived social expectations can shape women’s decision-making processes, potentially undermining their autonomy and reducing satisfaction with the birth experience.

Experts emphasized the importance of addressing these dynamics during childbirth preparation, encouraging women to reflect on how social norms and expectations, whether from healthcare providers, family, or broader cultural influences, might impact their choices, particularly regarding mobility during labor. This includes standard practices or clinical recommendations that, intentionally or not, may restrict movement or discourage upright positions. Introducing hypothetical scenarios during antenatal education was identified as a valuable strategy to foster critical thinking and support autonomous decision-making. By engaging in guided reflection and practicing how they might respond in specific situations, women can better recognize and navigate external pressures. For decision-making to be truly emancipated, aligned with personal values and conducive to satisfaction, women must become aware of how social expectations can create discomfort or conflict, and learn to make choices that prioritize their own needs and preferences^14^.

Despite robust evidence-based recommendations, the use of horizontal positions and restrictions on mobility during labor remain common in hospital settings^9,27^. A study conducted in Denmark found that although most women preferred to give birth in positions that allow for sacral flexibility, over 80% ultimately delivered in non-flexible sacrum positions, and fewer than half achieved their preferred birth position. Notably, self-efficacy levels did not increase the likelihood of attaining the desired position, suggesting that institutional culture and healthcare professionals’ attitudes play a critical role in shaping women’s choices during labor^27^.

Experts acknowledged the frustration women experience when faced with rigid hospital environments that prevent them from having the birth they envisioned. This frustration is shared by the professionals who accompany them throughout childbirth preparation but are unable to ensure those preferences are respected during labor. Providing woman-centered care remains a fundamental principle of midwifery. Similar findings were reported in a study from Australia, where midwives expressed a strong desire to ‘be with the women’, empowering them to make informed choices that align with their needs and circumstances, ultimately contributing to more positive birth experiences and better outcomes for both women and their families^34^.

In Portugal, where the public healthcare system does not guarantee a midwifery-led continuity of care model, experts emphasized the need for regular coordination between nurse-midwives working in community care units, who offer free childbirth preparation, and those in hospital labor wards. Regular meetings between these professionals could help align educational content with clinical practices and improve the likelihood of meeting women’s expectations. This recommendation is supported by a recent Cochrane review, which found that midwifery-led continuity of care is associated with reduced rates of cesarean sections, instrumental births, and episiotomies, and with increased rates of spontaneous vaginal births. In addition, this model improves women’s experiences across pregnancy, labor, and the postpartum period, while also reducing prenatal and intrapartum healthcare costs^35^. Although the review did not find a significant effect on epidural use, a study conducted in Ireland reported approximately a 20% reduction in epidural uptake, particularly among multiparous women and those followed by a community midwifery team. This decrease was attributed to the implementation of the ‘Labor Hopscotch Framework’, an intervention developed by midwives to promote mobility and active engagement during labor^36^.

### Strengths and limitations

This novel study explores the perspectives of nurse-midwives, recognized experts in childbirth preparation, on the essential components of an intervention designed to empower women for active labor, with a specific focus on decision-making. As key implementers of childbirth education programs, nurse-midwives offer valuable system-level insights that reflect both clinical realities and women’s needs. Their contributions helped shape an intervention that is evidence-based, contextually grounded, and aligned with current healthcare structures. The inclusion of participants from diverse regions and with varied professional experience across primary care, hospital settings, and academia enriched the analysis and ensured a multifaceted understanding of the topic. The use of a focus group enabled in-depth discussion and the sharing of diverse individual insights. Although women’s perspectives are equally vital, they were explored in a separate study currently under review. Together, these complementary viewpoints contribute to a more comprehensive, woman-centered intervention design.

Nonetheless, several limitations must be considered. Group dynamics, such as pre-existing relationships among some participants, and differences in age or social background, may have influenced levels of openness during discussion, potentially shaping the data collected. While the online format facilitated broad geographical participation, it may have limited the spontaneity and richness of interaction typically observed in face-to-face settings. Finally, the transferability of findings depends on the extent to which healthcare systems and childbirth practices in other contexts resemble those examined in this study.

## CONCLUSIONS

This study revealed a strong consensus among experts on the value of the proposed intervention, highlighting that staying active during labor benefits labor progression, perinatal outcomes, and the overall birth experience for women and their partners. Understanding women’s previous experiences and expectations was seen as essential for tailoring support. Experts emphasized the importance of empowering women through knowledge and practical skills, ideally developed through experiential, constructive, and interactive learning. Providing opportunities for women to physically experience the benefits of mobility and upright positions was viewed as critical for building confidence and self-efficacy. In more restrictive care settings, addressing the pressure stemming from social or institutional norms is particularly important. Supporting women to reflect on their options, anticipate scenarios, and engage in decision-making based on their individual context fosters more autonomous and informed choices.

Such learning should occur in an informal, supportive environment that encourages social connection and partner involvement, which are known to enhance self-efficacy and satisfaction. Experts also emphasized the central role of nurse-midwives in fostering responsive, woman-centered care environments. Ensuring continuity of care through regular communication and collaboration among nurse-midwives across different settings was considered essential for maintaining consistency, improving outcomes, and enhancing the overall quality of the childbirth experience.

Finally, the findings underscore the need for further research into decision-making interventions, particularly in highly medicalized or restrictive clinical settings, to identify effective strategies that promote autonomy, empowerment, and woman-centered care.

## Data Availability

The data supporting this research are available from the authors on reasonable request.

## References

[1] WHO recommendations: Intrapartum care for a positive childbirth experience. WHO; 2018. Accessed May 23, 2035. https://www.ncbi.nlm.nih.gov/books/NBK513809/pdf/Bookshelf_NBK513809.pdf 30070803

[2] Simkin P. Just another day in a woman’s life? Women’s long-term perceptions of their first birth experience. Part I. Birth. 1991;18(4):203-210. doi:10.1111/j.1523-536x.1991.tb00103.x 1764149

[3] Hodnett ED. Pain and women’s satisfaction with the experience of childbirth: a systematic review. Am J Obstet Gynecol. 2002;186(5)(suppl):S160-S172. doi:10.1067/mob.2002.121141 12011880

[4] Downe S, Finlayson K, Oladapo OT, Bonet M, Gülmezoglu AM. What matters to women during childbirth: A systematic qualitative review. PLoS One. 2018;13(4):e0194906. doi:10.1371/journal.pone.0194906 29664907 PMC5903648

[5] Lawrence A, Lewis L, Hofmeyr GJ, Styles C. Maternal positions and mobility during first stage labour. Cochrane Database Syst Rev. 2013;2013(10):CD003934. doi:10.1002/14651858.CD003934.pub4 24105444 PMC11664456

[6] Miller S, Abalos E, Chamillard M, et al. Beyond too little, too late and too much, too soon: a pathway towards evidence-based, respectful maternity care worldwide. Lancet. 2016;388(10056):2176-2192. doi:10.1016/S0140-6736(16)31472-6 27642019

[7] Healy S, Humphreys E, Kennedy C. A qualitative exploration of how midwives’ and obstetricians’ perception of risk affects care practices for low-risk women and normal birth. Women Birth. 2017;30(5):367-375. doi:10.1016/j.wombi.2017.02.005 28279637

[8] O’Brien D, Butler MM, Casey M. The importance of nurturing trusting relationships to embed shared decision-making during pregnancy and childbirth. Midwifery. 2021;98:102987. doi:10.1016/j.midw.2021.102987 33761433

[9] Shorey S, Chan V, Lalor JG. Perceptions of women and partners on labor and birth positions: A meta-synthesis. Birth. 2022;49(1):19-29. doi:10.1111/birt.12574 34245040

[10] Skivington K, Matthews L, Simpson SA, et al. A new framework for developing and evaluating complex interventions: update of Medical Research Council guidance. Int J Nurs Stud. 2024;154:104705. doi:10.1016/j.ijnurstu.2024.104705 38564982

[11] Lopes MI, Vieira M, Cardoso A. Women’s empowerment for active labor: A qualitative study with nurse-midwives in antenatal education for childbirth. Eur J Midwifery. 2024;8(August):1-10. doi:10.18332/ejm/188117 PMC11339881 39175493

[12] Tong A, Sainsbury P, Craig J. Consolidated criteria for reporting qualitative research (COREQ): a 32-item checklist for interviews and focus groups. Int J Qual Health Care. 2007;19(6):349-357. doi:10.1093/intqhc/mzm042 17872937

[13] Graneheim UH, Lundman B. Qualitative content analysis in nursing research: concepts, procedures and measures to achieve trustworthiness. Nurse Educ Today. 2004;24(2):105-112. doi:10.1016/j.nedt.2003.10.001 14769454

[14] Wittmann-Price RA. Exploring the subconcepts of the Wittmann-Price theory of emancipated decision-making in women’s health care. J Nurs Scholarsh. 2006;38(4):377-382. doi:10.1111/j.1547-5069.2006.00130.x 17181087

[15] Nieuwenhuijze MJ, de Jonge A, Korstjens I, Budé L, Lagro-Janssen TL. Influence on birthing positions affects women’s sense of control in second stage of labour. Midwifery. 2013;29(11):e107-e114. doi:10.1016/j.midw.2012.12.007 23415350

[16] Akin B, Yurteri Türkmen H, Yalnız Dilcen H, Sert E. The Effect of Labor Dance on Traumatic Childbirth Perception and Comfort: A Randomized Controlled Study. Clin Nurs Res. 2022;31(5):909-917. doi:10.1177/10547738211030745 34229473

[17] Johansson M, Thies-Lagergren L. Swedish fathers’ experiences of childbirth in relation to maternal birth position: a mixed method study. Women Birth. 2015;28(4):e140-e147. doi:10.1016/j.wombi.2015.06.001 26164103

[18] Mueller CG, Webb PJ, Morgan S. The Effects of Childbirth Education on Maternity Outcomes and Maternal Satisfaction. J Perinat Educ. 2020;29(1):16-22. doi:10.1891/1058-1243.29.1.16 32021058 PMC6984379

[19] Lopes MI, Wittmann-Price RA. The Wittmann-Price Theory of Emancipated Decision-Making in Women’s Health Care: An Analysis Based on McEwen. Holist Nurs Pract. 2025;39(3):141-150. doi:10.1097/HNP.0000000000000704 39723830

[20] Serçekuş P, Değirmenciler B, Özkan S. Internet use by pregnant women seeking childbirth information. J Gynecol Obstet Hum Reprod. 2021;50(8):102144. doi:10.1016/j.jogoh.2021.102144 33848646

[21] Artieta-Pinedo I, Paz-Pascual C, Grandes G, Villanueva G; Ema Q Group. An evaluation of Spanish and English on-line information sources regarding pregnancy, birth and the postnatal period. Midwifery. 2018;58:19-26. doi:10.1016/j.midw.2017.12.002 29277038

[22] Spiby H, Stewart J, Watts K, Hughes AJ, Slade P. The importance of face to face, group antenatal education classes for first time mothers: A qualitative study. Midwifery. 2022;109:103295. doi:10.1016/j.midw.2022.103295 35364368

[23] Wright A, Elcombe E, Burns ES. “Paper, face-to-face and on my mobile please”: A survey of women’s preferred methods of receiving antenatal education. Women Birth. 2021;34(6):e547-e556. doi:10.1016/j.wombi.2020.10.014 33172801

[24] Borer H, Dubovi I. Fostering childbirth education on upright positions and mobility during labor in nulliparous women. BMC Pregnancy Childbirth. 2023;23(1):870. doi:10.1186/s12884-023-06166-4 38104069 PMC10724979

[25] Demirci AD, Merve K, Kamile K. Effect of antenatal education on childbirth self-efficacy: A systematic-review and meta-analysis. Curr Psychol. 2023;42:11367-11377. doi:10.1007/s12144-021-02418-8

[26] Campbell V, Nolan M. ‘It definitely made a difference’: A grounded theory study of yoga for pregnancy and women’s self-efficacy for labour. Midwifery. 2019;68:74-83. doi:10.1016/j.midw.2018.10.005 30396001

[27] Kjeldsen LL, Dahlen HG, Maimburg RD. Expectations of the upcoming birth - A survey of women’s self-efficacy and birth positions. Sex Reprod Healthc. 2022;34:100783. doi:10.1016/j.srhc.2022.100783 36244077

[28] Feeley C, Thomson G, Downe S. Understanding how midwives employed by the National Health Service facilitate women’s alternative birthing choices: Findings from a feminist pragmatist study. PLoS One. 2020;15(11):e0242508. doi:10.1371/journal.pone.0242508 33216777 PMC7678977

[29] Cutajar L, Miu M, Fleet JA, Cyna AM, Steen M. Antenatal education for childbirth: Labour and birth. Eur J Midwifery. 2020;4(Arpil):1-9. doi:10.18332/ejm/120002 33537613 PMC7839135

[30] Bagnis A, Meeuwis SH, Haas JW, et al. A scoping review of placebo and nocebo responses and effects: insights for clinical trials and practice. Health Psychol Rev. 2025;19(2):409-447. doi:10.1080/17437199.2025.2471792 40028813

[31] Carlsson IM, Hallberg LR, Odberg Pettersson K. Swedish women’s experiences of seeking care and being admitted during the latent phase of labour: a grounded theory study. Midwifery. 2009;25(2):172-180. doi:10.1016/j.midw.2007.02.003 17600602

[32] Butler MM. Exploring the strategies that midwives in British Columbia use to promote normal birth. BMC Pregnancy Childbirth. 2017;17(1):168. doi:10.1186/s12884-017-1323-7 28583159 PMC5460538

[33] Eri TS, Blystad A, Gjengedal E, Blaaka G. ‘Stay home for as long as possible’: midwives’ priorities and strategies in communicating with first-time mothers in early labour. Midwifery. 2011;27(6):e286-e292. doi:10.1016/j.midw.2011.01.006 21454000

[34] Bloxsome D, Bayes S, Ireson D. “I love being a midwife; it’s who I am”: A Glaserian Grounded Theory Study of why midwives stay in midwifery. J Clin Nurs. 2020;29(1-2):208-220. doi:10.1111/jocn.15078 31633845 PMC7328794

[35] Sandall J, Fernandez Turienzo C, Devane D, et al. Midwife continuity of care models versus other models of care for childbearing women. Cochrane Database Syst Rev. 2024;4(4):CD004667. doi:10.1002/14651858.CD004667.pub6 38597126 PMC11005019

[36] Carroll L, Thompson S, Coughlan B, et al. ‘Labour Hopscotch’: Women’s evaluation of using the steps during labor. Eur J Midwifery. 2022;6:59. doi:10.18332/ejm/152492 36132188 PMC9460932

